# Enhancing meat-type chicken performance through *Thymus vulgaris* leaf powder supplementation by affecting serum lipid profile, stress physiology, immunity, antioxidants, cecal microbiology, and jejunal histomorphology

**DOI:** 10.5194/aab-68-311-2025

**Published:** 2025-05-21

**Authors:** Mohammad T. Banday, Manzoor Wani, Fatmah M. Alqahtani, Lovita Adriani, Majid Alhomrani, Sheikh Adil, Walaa F. Alsanie, Abdulhakeem S. Alamri, Osama Abdulaziz

**Affiliations:** 1 Division of Livestock Production and Management, Faculty of Veterinary Sciences and Animal Husbandry, SKUAST-K, Srinagar, India; 2 Department of Biology, College of Science, King Khalid University, Abha, 61413, Saudi Arabia; 3 Animal Husbandry Faculty, Padjadjaran University, Sumedang, Indonesia; 4 Department of Clinical Laboratory Sciences, The Faculty of Applied Medical Sciences, Taif University, Taif, Saudi Arabia; 5 Research center for health sciences, Deanship of Graduate Studies and Scientific Research, Taif University, Taif, Saudi Arabia; 6 Department of clinical laboratory science, College of Applied Medical Sciences, Taif University, Taif, Saudi Arabia

## Abstract

This study investigates the potential of *Thymus vulgaris* as feed additive in broiler chicken. A total of 200 Vencobb male broiler chicks 1 week of age were randomly distributed into 4 dietary treatments: T1 (control) fed basal diet only, T2 (basal diet +1 % thyme powder), T3 (basal diet +1 .5 % thyme powder), and T4 (basal diet +2 % thyme powder). Each group contained 5 replicates, and each replicate had 10 birds fed with a maize–soybean-based diet for 42 d. Our results show that during the overall period (7–42 d), the T4 group exhibited significantly ( p<0.05 ) higher body weight gain (BWG) and the lowest feed conversion ratio (FCR). Supplementing thyme powder significantly ( p<0.05 ) decreased the levels of blood cholesterol and triglycerides (LDL) compared to the control group. Birds receiving dietary thyme powder at a 2 % dose (T4) had significantly ( p<0.05 ) raised serum levels of immunoglobulin G (IgG) and immunoglobulin M (IgM) and better anti-SRBC (sheep red blood cell) titre and cell-mediated immunity. Supplementation of thyme powder resulted in significantly ( p<0.05 ) better antioxidant status in birds. Regarding the cecal microbiology, coliforms decreased ( p<0.05 ) and lactobacilli increased ( p<0.05 ) notably in thyme groups with high significance in the T4 group. Jejunal villus height increased significantly ( p<0.05 ) in the T4 group compared to the control. In conclusion, supplementation of thyme powder at 2 % could serve as promising feed additive for improving the production performance and gut health of broiler chicken.

## 1 Introduction

Dietary interventions, environment manipulations, and genetic advancements have all contributed to increased poultry performance (Rashid et al., 2017; Khurshid et al., 2019). Since more than 50 years ago, antibiotics have been used in poultry farming to enhance the general health and growth of the birds. Nevertheless, this use has led to a number of negative outcomes, including gut dysbiosis, loss of beneficial bacteria, antibiotic resistance, and possibility of disease transmission to humans (Ahmad et al., 2022). Bacteria can acquire antibiotic resistance from other bacterial species in the environment or by altering antibiotic binding sites and enzyme production or lowering the permeability of cell membranes (Christaki et al., 2020). Antibiotic-resistant bacteria are becoming more and more common in farm animals around the world, which both directly and indirectly contributes to the rise in human diseases brought on by these bacteria (Reddy and Saier, 2020). Many nations have restricted the use of antibiotics as growth promoters (AGPs) in order to address this worldwide public health concern and have also encouraged the development of antibiotic substitutes in veterinary and human medicine (Rahman et al., 2022). In the US and Europe alone, antimicrobial-resistant bacteria are responsible for about 50 000 fatalities annually (Shallcross and Davies, 2014).

Due to public health concerns and consumer demand, several antibiotic alternatives have been tested to enhance performance and health of poultry birds. Some of these alternatives include prebiotics, essential oils, plant extracts, organic acids, enzymes, and aquatic weeds (Adil et al., 2011; Yousuf et al., 2012; Untoo et al., 2018; Ara et al., 2018; Adil et al., 2020; Zaffer et al., 2021; Banday et al., 2024a, b). Currently, among numerous possibilities, phytogenic feed additives (PFAs) have received a lot of attention (Khurshid et al., 2016; Qureshi et al., 2016c; Adil et al., 2023, 2024a, b). PFAs can be used in feeds in several forms, including dry, solid, and powdered, as well as extracts (Gadde et al., 2017). Various PFAs in the form of herbs, spices, and essential oils have been used either separately or in combination, free or encapsulated, in poultry birds as prospective substitutes for AGPs (Wati et al., 2015; Adil et al., 2024a, b). The addition of PFAs to the diet has been shown to have a number of beneficial impacts on broiler performance, including improved gut health, positive modulation of gut microbiota dynamics, and increased feed efficiency (Qureshi et al., 2015, 2016a, 2017; Adil et al., 2024a, b).

Thyme (*Thymus vulgaris*), an aromatic Lamiaceae herb, is known for its antibacterial and antioxidant capabilities (Nameghi et al., 2019). Thyme's primary components are thymol (40 %) and carvacrol (15 %), which are its main bioactive compounds (Azaz et al., 2004). In their comparison of the effects of 60, 100, and 200 mg kg^−1^ dietary thymol and carvacrol supplementation in broiler performance, authors discovered a linear rise in average body weight growth with increasing supplementation levels (Hashemipour et al., 2013). Adding thyme powder to broiler chickens' diets increased growth performance and immunological status (Hassan and Awad, 2017). Broiler diets treated with 1 g kg^−1^ thyme powder resulted in significantly higher body weight gain than diets without supplements (Ragaa et al., 2016). Based on the beneficial qualities of the thyme powder, this study was taken up to look into the effect of thyme powder as a feed supplement on growth performance, blood indices, immuno-antioxidant parameters, and intestinal microbiology and microstructure of broiler chicken.

## 2 Materials and methods

### 2.1 Procurement of thyme and its analysis

The Faculty of Forestry at SKUAST-Kashmir, India, provided thyme plant. The leaves were collected and sun-dried in a shaded area. The resultant material was then ground into a fine powder in the Analytical Laboratory of Division of LPM, SKUAST-Kashmir, India. The proximate analysis of the samples was evaluated using the Association of the Official Analytical Chemists (AOAC) method (AOAC, 2005). Samples were dried in a hot-air oven set to 100 °C until a consistent weight was achieved in order to calculate the dry matter (DM), crude protein (CP) was calculated by the Kjeldahl method ( N×6.25 ) using a KEL Plus Nitrogen analyzer (Pelican, India), ether extract (EE) was determined by the Soxhlet extraction method using the SOCS Plus Automatic Solvent/Fat Extraction system (Pelican, India), crude fiber (CF) was calculated by standard procedure using FIBRA Plus Automatic Digestion System (Pelican, India), and total ash (TA) was found by incineration in a muffle furnace at 600 °C for 5 h. Total carbohydrates were calculated as nitrogen-free extract (NFE) (%) =100 % (moisture + CP + EE + CF + TA). All the readings were taken in triplicates and averaged.

**Table 1:** Ingredients and chemical composition of basal broiler chicken diet.

Ingredients	Starter diet	Finisher diet
Maize	57.28	60.51
Soybean	31.65	30.11
Fish meal	5.36	2.58
Soybean oil	2.89	3.67
Limestone	0.76	0.82
DCP	1.25	1.56
Salt	0.30	0.30
DL-meth	0.11	0.10
Lysine	0.05	0.00
TM premix^a^	0.10	0.10
Vitamin premix^b^	0.15	0.15
Choline chloride	0.05	0.05
Toxin binder	0.05	0.05
Total	100.00	100.00
Nutrient composition		
Crude protein	21.70	19.89
M energy	3068.84	3156.88
Calcium	1.00	1.00
Available P	0.45	0.45
Lysine	1.20	1.04
Methionine	0.50	0.45

^a^ Trace mineral premix (mg kg^−1^ diet): 100 Mn, 1.2 I, 80 Fe, 80 Zn, 0.15 Se, and 12 Cu. ^b^ Vitamin premix (per kg diet): 11 000 IU vitamin A, 3000 IU vitamin D3, 1.5 mg vitamin K, 30 mg vitamin E, 2.5 mg vitamin B1, 6 mg vitamin B2, 0.015 mg vitamin B12, 40 mg niacin, 15 mg pantothenic acid, and 500 mg choline. DCP: dicalcium phosphate, DL-meth: DL-methionine, TM premix: mineral premix, M energy: metabolizable energy.

### 2.2 Birds and experimental details

A total of two hundred 1 d old male Vencobb broiler chicks (average body weight 43±2.7 g) were procured and kept at the experimental facility of the Division of LPM, SKUAST, Kashmir, India. The chicks were raised together for 1 week before being weighed and randomly assigned to 20 floor pens with sawdust as litter material. The experimented lasted until the birds achieved 42 d of age. The experiment followed a completely randomized design, with four treatments and five replications (10 birds per replicate). Four diets, T1 (Control) fed basal diet only, T2 (basal diet +1 % thyme powder), T3 (basal diet +1 .5 % thyme powder), and T4 (basal diet +2 % thyme powder), based on a maize–soybean combination, were developed. National Research Council (1994) recommendations were followed in the formulation of the basal diet fed to broiler chicken. The ingredient and nutrient composition of the basal diet is presented in Table 1. The vaccination schedule and biosecurity measures were strictly followed, and the experiment was conducted in a very hygienic environment. The house's original temperature was maintained at 33 °C, and then it was lowered by 3 °C per week until it reached 24 °C. The birds were given a photoperiod that included 23 h of light and 1 h of darkness until the trial was over. The birds had unlimited access to feed and fresh water.

### 2.3 Production performance

Every week, the body weight (BW) and feed intake (FI) of each replicate were recorded. For the starter (7–21 d), finisher (22–42 d), and whole duration (7–42 d), body weight gain (BWG) and FI were calculated. Similarly, cumulative feed consumption was divided by cumulative BWG for the same periods to calculate the feed conversion ratio (FCR), which was then adjusted for mortality.

### 2.4 Sample collection

Two birds per replicate were chosen at random for slaughter at the end of the experiment (the 42nd day), and blood samples (for biochemistry, physiological stress, immunology, and antioxidant analysis) were collected from these birds after slaughtering following the halal procedure. Cecal contents were gathered into sterile polybags and kept at -20 °C until analysis for microbial count. Samples of the jejunum (extending from the end of the duodenum to the Meckel's diverticulum) measuring about 2 cm in length were cut with sterile scissors, cleaned in physiological saline to get rid of all the contents, and then preserved in 10 % buffered formalin solution for further histomorphological analysis.

### 2.5 Blood biochemistry

The blood-containing tubes were kept in an incubator at 37 °C for 1 h. The clots formed were broken, and the tubes were then centrifuged at 3000 RPM for half an hour. The serum was carefully transferred into smaller tubes using a pipette and stored at -20 °C until analysis. Various parameters such as total protein, cholesterol, triglycerides, alanine aminotransferase (ALT)-2CS3PTS180, aspartate aminotransferase (AST)-2CS3OTS180, and creatinine-2CS6CMR100 were analyzed using specific biochemical kits utilizing auto-analyzer equipment (Csense 200^CD^, Medsource Ozone Biomedicals Pvt. Ltd.).

### 2.6 Physiological stress

The physiological stress levels of birds were computed by determination of serum cortisol (CORT) and estimating the heterophil : lymphocyte (H : L) ratio. A commercial cortisol ELISA kit (Calbiotech Inc., USA) was used to evaluate the serum cortisol level. Further, the H : L ratio was calculated (Vleck et al., 2000). A clean glass slide was smeared with 5 µL of blood. After air drying and staining with the Wright–Giemsa method, the smears were seen under an oil-immersed microscope at a magnification of 100× . Differential counts were done, and heterophils and lymphocytes were counted in each visual area until the total number of cells reached 100, and, accordingly, the H : L ratio was calculated.

### 2.7 Immune study

ELISA kits were used to measure the level of total serum immunoglobulins IgG (CK-bio-18160) and IgM (CK-bio-18163) in accordance with the manufacturer's (Shanghai, China) recommended methods.

The Corrier and DeLoach (1990) approach was used to study the cell-mediated response to PHA-P obtained from Sigma-Aldrich. On the 22nd day of their life, 0.1 mL (1 mg mL^−1^) of PHA-P was injected into the third and fourth interdigital spaces of the right foot of the birds. The left foot received 0.1 mL of phosphate buffer and served as the control. The difference between the right and left foot's swelling before and after a 24 h injection was used to calculate the foot web index. It was computed as cell-mediated immunity (CMI) = (R2 - R1) - (L2 - L1) and measured in millimeters (mm), where L2 is the thickness following a 24 h phosphate buffer saline (PBS) injection, L1 is the thickness prior to a PBS injection, R2 is the thickness following a 24 h PHA-P injection, and R1 is the thickness prior to PHA-P injection.

Moreover, the anti-SRBC titre was measured using eight birds per treatment. The birds were injected with 0.1 % sheep red blood cells (SRBCs) at 0.1 mL kg^−1^ body weight into the veins in their wings at the age of 28 d. Following the outlined protocol (Akhlaghi et al., 2013), the blood samples were taken from the injected birds at 35 d of age, serum was extracted, and anti-SRBC antibody titre was measured using a 96-well microplate microhemagglutination assay.

### 2.8 Antioxidant study

The methods of Hadwan (2018), Kakkar et al. (1984), and Bradford (1976) were used to evaluate the serum antioxidant enzyme levels of catalase (CAT), superoxide dismutase (SOD), and malondialdehyde (MDA), respectively.

### 2.9 Cecal microbiology

For the aim of examining the microbiological characteristics, 1 g of each cecal sample was diluted 10^−1^ in 9 mL of sterile saline peptone solution and stirred for half an hour. Serial 10-fold dilutions up to 10^−6^ were made in pre-sterilized tubes. We purchased various media from HiMedia Laboratories Pvt. Ltd. for particular bacteria. A 0.1 mL aliquot of each dilution was distributed across a variety of specialized media, including DeMan–Rogosa–Sharpe agar for lactobacilli (37 °C for 48 h), MacConkey agar for coliforms (37 °C for 24 h), and plate count agar for total bacterial count (TBC) (30 °C for 48 h). The counts were reported as ${\mathrm{log}}_{10}$ colony-forming units per gram (cfu g^−1^) in accordance with Speck (1984).

### 2.10 Histomorphological study

After being dried, samples of jejunum treated in 10 % formalin were embedded in paraffin wax. Sections of the paraffin-embedded tissue blocks (6 µm thick) were then cut out using a microtome and stained with hematoxylin and eosin. The stained tissues were examined using a Nikon Eclipse Ni, DS-Ri2 microscope fitted with a computer-assisted morphometric system. The mean of 10 randomly chosen sections from each sample was used to calculate the crypt depth (CD) and villus height (VH) and subsequently the VH-to-CD ratio (Thompson and Applegate, 2006).

### 2.11 Statistical analysis

We utilized one-way ANOVA with a 5 % significance level to analyze variances. Duncan's multiple range test was used to assess the significance of variance across groups. All statistical tests were conducted using SPSS software (version 20.0).

**Figure 1:**
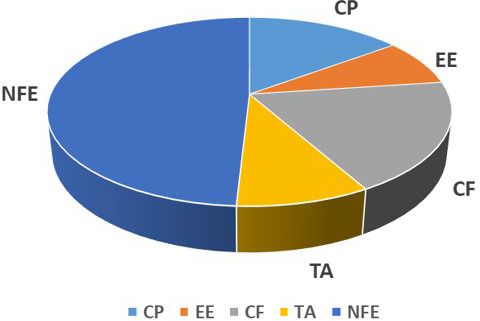
Proximate composition of thyme powder. CP: crude protein, EE: ether extract, CF: crude fiber, TA: total ash, NFE: nitrogen-free extract.

## 3 Results

### 3.1 Nutritive value of thyme powder

The results related to proximate analysis of thyme powder are illustrated in Fig. 1. The thyme powder contained DM (91.62 %), CP (13.57 %), EE (7.38 %), CF (17.41 %), TA (8.23 %), and NFE (45.03 %).

**Table 2:** Effect of thyme powder supplementation on the performance of broiler chicken at different stages of production.

Parameter	T1	T2	T3	T4	SEM	P value
BWG (g)						
1–3 weeks	498.16^c^	511.87^b,c^	533.52^a,b^	552.88^a^	7.37	0.012
4–6 weeks	1356.78^b^	1377.74^a,b^	1384.99^a^	1392.80^a^	5.27	0.035
1–6 weeks	1854.94^c^	1889.61^b,c^	1918.51^a,b^	1945.67^a^	11.65	0.007
FI (g)						
1–3 weeks	608.39	614.90	621.74	625.86	4.22	0.537
4–6 weeks	2565.95	2566.77	2546.59	2551.14	9.55	0.874
1–6 weeks	3174.33	3181.67	3168.33	3177.00	11.48	0.987
FCR						
1–3 weeks	1.22^b^	1.20^a,b^	1.17^a,b^	1.13^a^	0.013	0.008
4–6 weeks	1.89^b^	1.86^a,b^	1.84^a,b^	1.83^a^	0.010	0.047
1–6 weeks	1.71^b^	1.68^b^	1.65^a,b^	1.63^a^	0.011	0.025

Means with different superscripts in same row differ significantly ( P<0.05 ). T1: control, T2: basal diet +0.5 % thyme powder, T3: basal diet +1 % thyme powder, T4: basal diet +2 % thyme powder. BWG: body weight gain, FI: feed intake, FCR: feed conversion ratio, SEM: standard error of the mean.

### 3.2 Effect on growth performance

Table 2 displays the impact of various dietary treatments on the performance of broiler chicken. BWG of birds showed a significant ( p>0.05 ) effect between dietary treatments and control during starter (weeks 1–3) and finisher (weeks 4–6) periods. There was a significant treatment effect on BWG when compared with the control group during the overall period (weeks 1–6). the lowest BWG was found in the control (1854.94 g/bird) and tended to become better in all other treatments. The highest improvement of 4.66 % in the BWG was recorded in the T4 group that was fed 2 % thyme powder in the diet when compared to control ( P=0.007 ). There was no significant ( p>0.05 ) difference observed in FI among the dietary treatments and the control group. Overall (7–42 d), FCR reduced significantly ( P=0.025 ) in the T4 group when compared to other treatments and the control. The T2 and T3 groups showed no significance ( p>0.05 ) in the FCR when compared to the control.

**Table 3:** Effect of thyme powder supplementation on blood biochemistry and stress physiology of broiler chicken at 42 d of age.

Parameter	T1	T2	T3	T4	SEM	P value
TP (g dL^−1^)	4.73	4.65	4.76	4.95	0.05	0.221
Cholesterol (mg dL^−1^)	163.80^a^	161.89^a^	138.53^b^	136.21^b^	4.63	0.019
Triglyceride (mg dL^−1^)	106.51^a^	102.94^a,b^	98.39^a,b^	86.57^b^	2.95	0.048
ALT (U mL^−1^)	110.79	113.07	106.22	108.91	2.50	0.844
AST (U mL^−1^)	16.10	17.34	15.68	14.26	0.62	0.421
Creatinine (mg dL^−1^)	0.39	0.42	0.37	0.40	0.02	0.846
Cortisol	63.48	67.20	55.82	49.31	2.89	0.627
H : L ratio	0.61	0.56	0.58	0.52	0.02	0.539

Means with different superscripts in same row differ significantly ( P<0.05 ). T1: control, T2: basal diet +0.5 % thyme powder, T3: basal diet +1 % thyme powder, T4: basal diet +2 % thyme powder. TP: total protein, ALT: alanine aminotransferase, AST: aspartate aminotransferase, H : L ratio: heterophil : lymphocyte ratio, SEM: standard error of the mean.

### 3.3 Effect on blood biochemical and stress physiology

The results of blood biochemicals and stress physiology in broiler chickens at 42 d of age are displayed in Table 3. Among all the treatments, including the control, the addition of thyme powder did not result in significant ( p>0.05 ) changes in the levels of total protein, alanine transaminase (ALT), aspartate aminotransferase (AST), or creatinine. In contrast to T2, T3, and control, thyme powder added to the diet of broilers at a 2 % level (T4) resulted in a significant ( p<0.05 ) decrease in serum cholesterol and triglyceride levels.

The results revealed that there was a numerical reduction in the physiological stress of birds in terms of lower serum CORT and H : L ratio in the group that was fed 2 % thyme powder in the diet when compared to other dietary treatments and the control.

**Figure 2:**
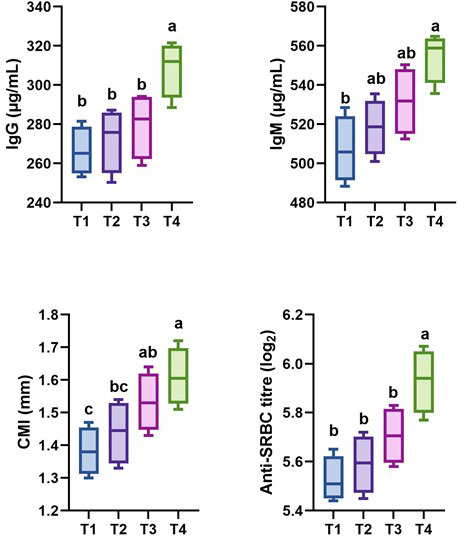
Effect of thyme powder supplementation on immunity of broiler chicken at 42 d of age. IgG: immunoglobulin G, IgM: immunoglobulin M, CMI: cell-mediated immunity, anti-SRBC titre: anti-sheep red blood cell titre. Different letters (a, b, and c) indicate significant differences ( p<0.05 ), while the same letter indicates no significant differences ( p>0.05 ). T1: control, T2: basal diet +0.5 % thyme powder, T3: basal diet +1 % thyme powder, T4: basal diet +2 % thyme powder.

### 3.4 Effect on immune parameters

The results of supplementing broilers with thyme powder on their immunological parameters at 42 d of age are presented in Fig. 2. In comparison to the other groups and the control, the serum IgG and IgM values increased significantly ( p<0.05 ) in the T4 group. CMI was found to be significantly ( p<0.05 ) better in T3 and T4 groups. The anti-SRBC titre was found to be significantly ( p<0.05 ) improved in T4 when compared to the control and other dietary treatments.

**Figure 3:**
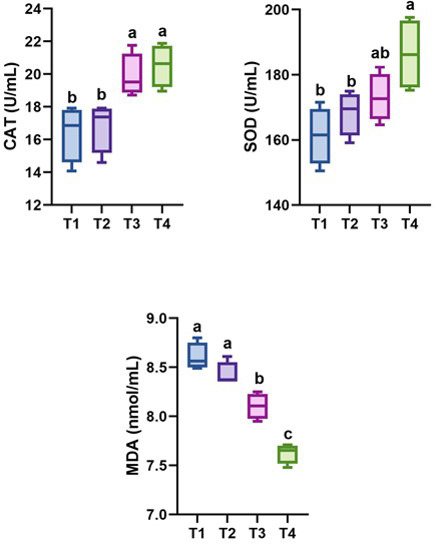
Effect of thyme powder supplementation on serum antioxidants of broiler chicken at 42 d of age. CAT: catalase, SOD: superoxide dismutase, MDA: malondialdehyde. Different letters (a, b, and c) indicate significant differences ( p<0.05 ), while the same letter indicates no significant differences ( p>0.05 ). T1: control, T2: basal diet +0.5 % thyme powder, T3: basal diet +1 % thyme powder, T4: basal diet +2 % thyme powder.

### 3.5 Effect on serum antioxidants

Figure 3 depicts the impact of dietary thyme powder supplementation on serum antioxidant levels at 42 d of age. Broiler birds supplemented with thyme powder at the 2 % level (T4) had significantly ( p<0.05 ) higher values for catalase and SOD values in comparison to the control group. Further, the levels of MDA decreased ( p<0.05 ) in birds belonging to the T4 group when compared to the birds in rest of the groups.

**Table 4:** Effect of thyme powder supplementation on cecal microbiology of broiler chicken at 42 d of age.

Parameter (log_10_ CFU g^−1^)	T1	T2	T3	T4	SEM	P value
TBC	8.94	8.93	8.87	8.81	0.024	0.230
Coliforms	6.37^a^	6.35^a^	6.28^a,b^	6.17^b^	0.025	0.007
Lactobacilli	4.84^b^	4.87^b^	4.91^a,b^	4.98^a^	0.019	0.032

Means with different superscripts in the same row differ significantly ( P<0.05 ). T1: control, T2: basal diet +0.5 % thyme powder, T3: basal diet +1 % thyme powder, T4: basal diet +2 % thyme powder. TBC: total bacterial count, SEM: standard error of the mean.

### 3.6 Effect on cecal microbiology

Table 4 shows the effects of thyme powder supplementation on the cecal bacteria of broiler chicks at 42 d of age. The TBC of broilers was not affected ( p>0.05 ) by the inclusion of thyme powder up to the 2 % level in the diet when compared to the control. Inclusion of thyme powder at the 2 % level in the diet significantly ( p<0.05 ) decreased the coliform count when compared to the control birds. The *Lactobacillus* count increased significantly ( p<0.05 ) in the T4 group when compared to other dietary treatments and control.

**Figure 4:**
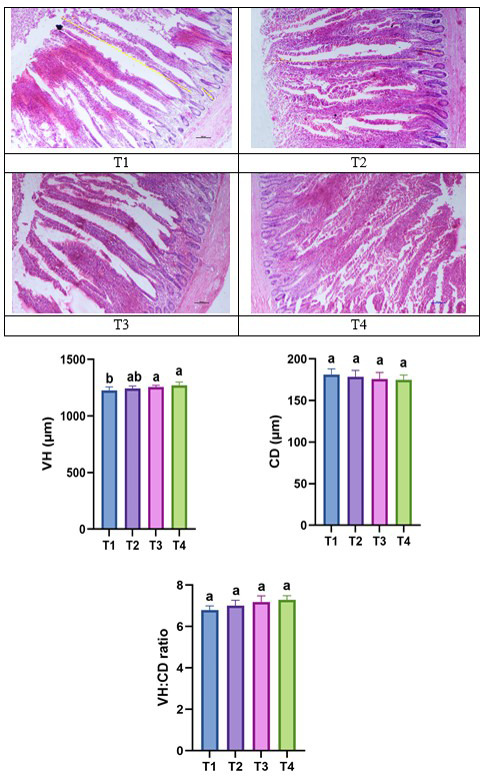
Photomicrographs showing the effect of thyme powder supplementation on jejunal histomorphology of broiler chicken at 42 d of age. VH: villus height, CD: crypt depth, VH : CD ratio: villus height : crypt depth ratio. T1: control, T2: basal diet +0.5 % thyme powder, T3: basal diet +1 % thyme powder, T4: basal diet +2 % thyme powder.

### 3.7 Effect on intestinal microstructure

Figure 4 shows the impact of dietary supplementation of thyme powder on jejunal histomorphology of broilers at 42 d of age. The data revealed that the diets supplemented with 1.5 % and 2 % thyme powder resulted in significantly ( p<0.05 ) better VH in the jejunum when compared to the control group. CD and VH : CD ratio of broiler birds showed no statistical difference among various dietary treatments and control.

## 4 Discussion

The proximate analysis of thyme powder revealed 91.63 % DM, 13.57 % CP, 7.38 % EE, 17.41 % CF, 8.24 % TA, and 45.03 % NFE. More or less similar results were documented earlier by authors (El-Refai et al., 2020) who reported CP, EE, CF, TA, and NFE of thyme powder to be 9.67 %, 12.46 %, 7.18 %, and 61.4 %, respectively. Further, the proximate composition of thyme was reported to be 93.14 % DM, 14.15 % CP, 6.86 % EE, 16.23 % CF, and 10.03 % TA (Biel et al., 2023). However, contrary to our results, others (Abdel-Wareth et al., 2012) reported a higher value for CP (19.30 % and) and lower values for EE (6.10 %) and TA (6.51 %). This variation in the results could be due to the various species of thyme utilized and environmental factors, such as soil fertility, moisture content, and growing temperature, that significantly impact the nutritional value of plants cultivated in different geographical regions.

The present study revealed that the supplementation of dietary thyme powder in birds improved their BWG and FCR particularly at the 2 % level without affecting the feed intake. The results of our study were in consistent with Abdel-Wareth et al. (2012), who reported an increase in BWG as a result of the quadratic effect of thyme powder at 1.5 % and 2 % levels in the diet of broiler chicken. Similarly, a significant increase in the body weight, BWG, and FCR of broilers with no effect on FI by adding up to 0.5 % of thyme to broiler diet has been noticed (Adam et al., 2020). An earlier study (El-Hakim et al., 2009) reported that thyme has an important role in the weight gain of broilers until the 21st day of age compared with control groups. Likewise, an improvement in the average daily weight gain and FCR of broilers that were fed a combination of thyme and rosemary powder when compared to the control has been found (Nameghi et al., 2022). These results are in concordance with our earlier studies (Adil et al., 2024a; Banday et al., 2024a, b). Adding 200 ppm of thyme extract to broilers' drinking water improved BWG and FCR when compared to control birds (Feizi and Bijanzad, 2010).

The improvement in the performance of birds that were fed thyme powder has been attributed to the various antioxidants and phenolic substances present in it, which can reduce the pathogenic microbial population of the gut and improve the absorption of various nutrients (Lee et al., 2003). Thymol and carvacrol, the two most significant bioactive substances found in this plant, may be the main causes of thyme's pharmacological effects (Grigore et al., 2010). Moreover, the bioactive components like carvacrol has been found to have a stimulatory effect on the secretion of digestive enzymes, thereby improving the digestion of various nutrients in the gut (Ragaa et al., 2016). Other authors (Manzanilla et al., 2001) also reported that beneficial antioxidant compounds of medicinal plants protect intestinal villi by improving nutrient uptake, thereby improving bird performance. On the basis of our results, we could attribute improvement in the BWG and FCR of birds to the beneficial effect of thyme powder on the lipid profile, reducing physiological stress, exerting positive impact on immune and antioxidant status, and having favorable impact on intestinal microbiology and microstructure.

In contrast to our study, some authors (Abdel-Ghaney et al., 2017) reported no improvement in the BWG and FCR of birds fed thyme powder up to 1.5 % in the diet. Likewise, an earlier study (Hosseini et al., 2016) found that BWG, FI, and FCR of broilers were not affected on supplementation of ground thyme at 0.5 % and 0.75 % in the diet compared to the control birds.

Serum biochemistry is a labile biochemical system which can measure the bird's physiological, nutritional, and pathological state and changes it experiences as a result of both internal and external influences. Therefore, the serum biochemical profile can be correlated to screen medicinal compounds and measure their impact, which will indicate whether or not they are suitable for a given species (Prashant et al., 2012).

Serum total protein (TP) concentration (g dL^−1^) obtained in the present study did not vary significantly on the supplementation of thyme powder up to 2 % in the diet, and all the values fall within the normal range (Campbell, 2004). Low protein levels can be a sign of liver damage or other disorders. Proteins are necessary for growth as well as many other processes, such as immunity and disease prevention. The results obtained in our study were in agreement with our earlier studies (Adil et al., 2024b; Banday et al., 2024b), where we reported no significant difference in serum total protein levels on addition of phytogenics in the diet. Moreover, no significant difference in TP in supplementation consisting of a combination of thyme powder and its extract in broilers was recorded (Belali et al., 2022).

Furthermore, in our study, a significant reduction in serum total cholesterol and triglycerides (TG) levels was observed in birds fed diets supplemented with thyme. In one more study, it was documented that supplementing broiler diet with 0.5 %, 1 %, or 1.5 % thyme-leaf powder decreased cholesterol level of birds (Ali, 2014). Moreover, a significant reduction in total cholesterol and TG levels upon addition of phytogenics in the diet has been found earlier (Adil et al., 2024b; Banday et al., 2024b). Thymol and carvacrol may have a hypocholesterolemic and antihyperlipidemic impact by inhibiting the rate-limiting enzyme of cholesterol, HMG-CoA reductase, which in turn decreased intestinal fat absorption or lipid catabolism for gluconeogenesis (Abdulkarimi et al., 2011). However, contrary to our results, no significant difference was found in total cholesterol and TG levels upon supplementation of a combination of thyme powder and its extract in broilers (Belali et al., 2022). Similarly, others also documented no significant difference in total cholesterol and TG levels upon addition of thyme powder up to the 0.1 % level (Toghyani et al., 2010).

Aspartate transaminase (AST) and alanine transaminase (ALT) activity are two crucial markers of a healthy liver (Ambrosy et al., 2015). Increased liver injury or malfunction is indicated by the elevated levels of these enzymes in the serum (Ileke et al., 2014). Our study revealed no significant change in both ALT and AST, which agrees with the results obtained by Ragaa et al. (2016) who reported no significant difference in liver enzymes. Nameghi et al. (2022) also found no change in ALT and AST levels upon addition of thyme powder up to the 0.75 % level, but, contrary to our results, others (Belali et al., 2022) reported a significant difference in alanine transaminase, lactate dehydrogenase, and creatine kinase of broilers fed different levels of thyme aqueous extract and thyme powder. Kidneys are the body's primary excretory organs, eliminating waste products from several metabolic processes and muscular contractions (Ileke et al., 2014). When diagnosing renal impairment, one of the most sensitive biochemical markers used is the serum creatinine level (Akande et al., 2013). Kidney injury is indicated by a serum creatinine level that is higher than usual. The values of serum creatinine obtained in our study revealed no significance in supplementation with thyme powder up to the 2 % level compared to the control, which agrees with Ragaa et al. (2016), who found no significance in creatinine levels upon supplementation of thyme or a combination of thyme and formic acid in the diet of birds.

Stress is defined as any condition that negatively affects an animal's biological processes (Uyanga et al., 2023). Extreme temperatures, rapid growth rates, overcrowding or high stocking density, malnutrition, and other factors can all produce physiological stress in animals (Qureshi et al., 2020). In order to measure the level of stress in birds, serum cortisol concentration is a commonly and frequently assessed biomarker of stress in birds (Weimer et al., 2018; Freeman and Newman, 2018; Will et al., 2019) besides heterophil : lymphocyte (H / L) ratio. Our results revealed a non-significant reduction in the serum cortisol concentration and H / L ratio as the level of thyme powder was increased, and maximum reduction was observed at the 2 % level. These findings are consistent with other studies (Olfati and Mojtahedin, 2018; Adil et al., 2024b; Banday et al., 2024a), which found that phytobiotic supplementation reduce heterophil count and H / L ratio, increase lymphocyte count, and are more effective in protecting chicks from the negative effects of heat stress. Phytobiotics can protect poultry birds from environmental stresses and boost their immune system (Adil et al., 2024b).

Our study revealed an increase in the serum concentration of IgG and IgM, which is consistent with our earlier studies (Adil et al., 2024b; Banday et al., 2024a, b), which report that dietary phytobiotic supplementation significantly increased the serum levels of IgG and IgM levels in broilers. However, contrary to our results, Toghyani et al. (2010) and Belali et al. (2022) reported that supplementation of thyme powder at the 1.5 % and 1 % level, respectively, had no significance in the immune response of broiler chicken, and this variation may be due to the dosage of the thyme and strain of the birds used in the experiment. The present study further indicates significant improvement in the cell-mediated immunity and anti-SRBC titre of birds supplemented with thyme powder. These results corroborate the results of Fallah and Mirzaei (2016), who observed that an addition of thyme powder of 1 g kg^−1^ diet increased antibody titres against Newcastle and influenza viruses. Ganesh and Bharat (2007) documented that as strong immunomodulatory agent, thyme powder can alter the activation of dendritic cells, T cells, B cells, neutrophils, natural killer cells, and macrophages. Additionally, herbs high in flavonoids, such as thyme, increase the action of vitamin C, work as antioxidants, and may therefore improve immunological function (Acamovic and Brooker, 2007).

CAT and SOD protect intracellular lipids from peroxidation, whereas MDA indicates the level of lipid peroxidation (Moustafa et al., 2020). There was a significant increase in the serum antioxidants such as CAT and SOD and a decrease in MDA levels upon supplementation with thyme, particularly at the 2 % level. These results agree with our previous findings (Adil et al., 2024b; Banday et al., 2024a), which report that serum SOD activity and glutathione (GSH) levels significantly increased in the groups that were fed diets supplemented with phytogenics. Cellular antioxidant activity is determined by the balance between reactive oxygen species (ROSs) produced and the intrinsic antioxidant defense system of the body (Burton and Jauniaux, 2011). According to an earlier study (Abdel-Ghaney et al., 2017), adding thyme to the broiler diet significantly raised SOD levels while lowering MDA levels in the muscles of the thigh and breast. It has been suggested that the high biological activity of thyme as a naturally occurring antioxidant results from the presence of phenolic hydroxyl groups, which prevent the formation of hydroxyl peroxide by acting as a hydrogen donor to the proxy radicals generated during the initial stage of lipid oxidation (Hashemipour et al., 2013).

A significant reduction in the cecal coliform count and an increase in lactobacilli count was recorded as the level of thyme powder was increased in the diet of birds. However, TBC did not vary between control and dietary treatments. These results are in agreement with other authors (Nameghi et al., 2022), who reported that the inclusion of thyme powders in broiler diets changes the gut microflora toward increasing lactobacilli and decreasing *Escherichia coli*. Similar results in support of our study were also documented by previous workers (Norouzi et al., 2015). Moreover, it was reported that birds fed 200 ppm thyme essential oil in drinking water had a higher ileal lactobacilli and a lower *E. coli* count compared to the control birds (Saki, 2014). The microbiome of the gastrointestinal tract has a role in pathogen defense, nutrition absorption, immune system maturation, and overall performance of birds. Poor gut health leads to food malabsorption and decreased development in birds (Bailey, 2010). Phytogenic substances are known to regulate gut microbiota, hence improving the hosts' health (Qureshi et al., 2016b; Adil et al., 2024b). Numerous studies have demonstrated that phytogenic substances or their extracts lower the population of harmful bacteria and their metabolites while increasing the growth of good microflora, which protects the bird against numerous diseases (Adil et al., 2024b; Banday et al., 2024a, b).

The small intestine absorption capacity can be assessed by the morphology of the villus and crypts, as well as the VH : CD ratio. Longer villi with more surface area can result in higher feed utilization, better growth performance, and improved bird health (Pluske et al., 1996). In our study, supplementation of thyme, especially at the 2 % level, resulted in a significant increase in the villus height of broilers as compared to the control birds. Phytogenic supplementation has been shown to improve gut microarchitecture by increasing villus height and surface area (Adil et al., 2024b; Banday et al., 2024a, b) and increase absorption and mucus secretion, hence reducing pathogen adherence in the intestines (Alcicek et al., 2004). Zeng et al. (2015) documented that thyme contains active compounds which can reduce intestinal infections by resisting pathogenic bacteria and increasing mature enterocytes, which improves villus height and absorption efficiency.

## 5 Conclusions

In conclusion, in the current study, body weight and feed efficiency of broiler birds improves in thyme-powder-supplemented groups, particularly in the 2 % thyme powder group. These results may be attributed to the beneficial effects of thyme powder supplementation on the lipid profile, immunological parameters, serum antioxidants, intestinal microbiology, and histomorphology. Based on the current findings, thyme powder at a concentration of 2 % could be utilized as a feed supplement to improve broiler chicken performance and gut health. Future research into potential synergies with other phytogenic additions may show complementary effects, while molecular investigations on microbiology, immunology, and antioxidant pathways may provide a better knowledge of thyme's biological effects.

## Data Availability

The data generated has been included in Figs. 1–4 and Tables 1–3.
